# Intra- and inter-exam reproducibility of left ventricular twist measurements using Fourier analysis of STimulated Echoes (FAST)

**DOI:** 10.1186/1532-429X-15-S1-E3

**Published:** 2013-01-30

**Authors:** Meral Reyhan, Hyun J Kim, Matthew S Brown, Daniel Ennis

**Affiliations:** 1 Department of Radiological Sciences, University of California, Los Angeles, CA, USA; 2 Biomedical Physics Interdepartmental Program, University of California, Los Angeles, CA, USA

## Background

Assessing the reproducibility of new quantitative magnetic resonance imaging (MRI) biomarkers is an important part of validation [1-3]. Fourier Analysis of STimulated Echoes (FAST) [4] is a new MRI tissue tagging method that has recently been shown to compare favorably to conventional estimates of left ventricular (LV) twist from cardiac tagged images, but with significantly reduced user interaction time (less than ~3 minutes) and low intra- and inter-observer variability. The purpose of this study was to assess the intra- and inter-exam reproducibility of LV twist using FAST.

## Methods

After obtaining informed consent, healthy volunteers (N=10) were examined on Day-1 and Day-8. On Day-1 MRI tagging was used to collect two measurements of LV twist for intra-exam comparisons. LV twist was measured again on Day-8 for inter-exam assessment. The following imaging parameters were used to acquire short-axis images at the base and apex: 280-330 x 280-330 mm field-of-view, 6mm slice thickness, 192x192 acquisition matrix, 395 Hz/pixel receiver bandwidth, TE/TR =2.33-2.39/4.71-4.83 ms, 8 VPS, 12° imaging flip angle, 8 mm tag spacing, i-pat acceleration factor 2 with 24 central lines, and 14-27 cardiac phases. The breath hold duration was 15 heart beats (12.5 ± 1.9 seconds) depending upon heart rate. LV short-axis tagged images were acquired on a 3T scanner in order to ensure detectability of tags during systole to mid-diastole. The collection order of horizontal and vertical tags at the apex and base of the LV was randomized for each exam. FAST was used to automatically estimate LV systolic and diastolic twist parameters subsequent to ~3 minutes of user interaction. Peak LV twist was reported as mean±SD. Reproducibility was assessed using the concordance correlation coefficient (CCC) and the repeatability coefficient (RC= 95%-CI range).

## Results

Mean peak twist measurements were 13.4±4.3° (Day-1, Exam-1), 13.6±3.7° (Day-1, Exam-2), and 13.0±2.7°(Day-8). Bland-Altman analysis resulted in intra- and inter-exam bias and 95%-CI of -0.6° [-1.0°, 1.6°] and 1.4° [-1.0°, 3.0°], respectively. Paired t-tests showed no significant differences in peak LV twist for intra- and inter-exam values (all p>0.1). The Bland-Altman RC for peak LV twist was 2.6° and 4.0° for intra- and inter-exam respectively. The CCC was 0.9 and 0.6 for peak LV twist for intra- and inter-exam respectively indicating excellent and moderate agreement respectively.

## Conclusions

FAST is a semi-automated method that provides a quick and quantitative assessment of LV systolic and diastolic twist that demonstrates excellent intra-exam and moderate inter-exam reproducibility in preliminary studies. FAST estimates of LV twist may serve as a useful biomarker of LV dysfunction in longitudinal studies.

## Funding

Funding from AHA and UCLA to MLR and NIH to DBE.

**Table 1:** 

	Mean Peak Twist	Bland-Altman Bias (95% CI)	RC	CCC	P-Value
Intra-Exam	13.4±4.3°,13.6±3.7°	-0.6° [-1.0°, 1.6°]	2.6°	0.9	P=0.9

Inter-Exam	13.4±4.3°,13.0±2.7°	1.4° [-1.0°, 3.0°]	4.0°	0.6	P=0.11
